# Assessing and Preparing Patients for Hematopoietic Stem Cell Transplant in Canada: An Environmental Scan of Psychosocial Care

**DOI:** 10.3390/curroncol30090617

**Published:** 2023-09-15

**Authors:** Sara Beattie, Maryam Qureshi, Jennifer Pink, Zen Gajtani, Andrea Feldstain

**Affiliations:** 1 Department of Psychosocial and Rehabilitation Oncology, Tom Baker Cancer Centre, Calgary, AB T2N 4N2, Canada; j3pink@uwaterloo.ca (J.P.); zgajtani@ucalgary.ca (Z.G.); andrea.feldstain@albertahealthservices.ca (A.F.); 2 Division of Psychosocial Oncology, Department of Oncology, Cumming School of Medicine, University of Calgary, Calgary, AB T2N 4N1, Canada; 3 Department of Counselling Psychology, Werklund School of Education, University of Calgary, Calgary, AB T2N 1N4, Canada; mquresh@ucalgary.ca

**Keywords:** hematopoietic stem cell transplant, environmental scan, psychoeducation, psychosocial screening, qualitative inquiry

## Abstract

Recipients and caregivers of Hematopoietic Stem Cell Transplant (HCT) have extensive physical and psychosocial needs. HCT programs recognize the need to support psychosocial wellbeing. However, evidence-based guidance for pre-HCT psychosocial services is sparse. We conducted a qualitative environmental scan of programs across Canada to better understand how programs evaluate and support patients and caregivers prior to HCT. Methods: HCT programs across Canada were contacted with a list of questions about their psychosocial assessment and preparation process with patients and caregivers. They could respond via email or participate in an interview over the phone. Descriptive qualitative content analysis was conducted, using steps outlined by Vaismoradi and colleagues (2013). Results: Most participants were social workers from hospitals (64%). Four qualitative themes arose: (a) Psychosocial Team Composition. Psychosocial assessment for HCT patients was often provided by social workers, with limited availability of psychologists and psychiatrists. (b) Criteria for assessing select HCT patients. Participants prioritized psychosocial assessments for patients with higher perceived psychosocial needs or risk, and/or according to transplant type. Limited time and high psychosocial staff demands also played into decision-making. (c) Components and Practices of Pre-HCT Psychosocial Assessment. Common components and differences of assessments were identified, as well as a lack of standardized tools. (d) Patient Education Sessions. Many sites provided adjunct patient education sessions, of varying depth. Conclusion: Significant variation exists in the way programs across the country assess their patients’ psychosocial pre-transplant needs and assist in preparing patients for the psychosocial aspects of HCT. This environmental scan identified several strategies used in diverse ways. Further in-depth research on program outcomes across Canada could help to identify which strategies are the most successful.

## 1. Introduction

Hematopoietic stem cell transplantation (“HCT,” also known as cellular therapy transplantation or blood and marrow transplantation) is a treatment for malignant and non-malignant conditions, primarily used for hematological cancers [1]. HCT involves a myeloablative process followed by the reintroduction of stem cells, harvested beforehand from either the patients themselves (autologous or “auto” HCT) or from a donor (allogeneic or “allo” HCT). HCT offers a potential cure for illnesses that are otherwise considered incurable. However, HCT may involve long hospital stays, the need to travel to distant centres for multiple months, dedicated family caregiver support, and an extensive recovery time with high morbidity [2,3,4,5]. As well, care is trending away from lengthy inpatient stays and towards outpatient care [6], requiring additional support from lay caregivers. These support demands may extend for multiple months or even beyond a year. Understandably, this process can lead to significant psychosocial morbidity for patients and their caregivers.

The most frequent unmet needs for HCT patients are psychosocial, including emotional, cognitive, social, and financial [7,8]. Patients and caregivers can experience psychological morbidity from the transplant process, including depression, posttraumatic stress disorder, sleep disorders, sexual dysfunction, and diminished quality of life. These negative outcomes may be exacerbated by longer hospitalizations [9,10], higher symptom burden, or psychosocial factors such as unemployment, being female, lower education, or avoidant coping [11,12]. Quality psychosocial screening may identify risk factors early and facilitate a referral to appropriate services, such as psychology, social work, or psychiatry, to bolster psychosocial outcomes throughout the transplant process. A description of psychosocial clinicians and roles in supporting patients and caregivers through HCT can be found in Buchbinder and Khera’s manuscript [8].

Screening and referral practices that consider psychosocial risk factors and the need for additional professional supports have the potential to improve post-transplant psychosocial morbidity and survival for patients and caregivers [6]. Previous literature recognizes that many centres evaluate pre-transplant psychosocial factors to some extent [8]. However, little evidence exists to inform best practice: only one study surveyed the practices of psychosocial assessment across US centres [13].

The need exists for HCT programs to support the psychosocial wellbeing of their patients and caregivers, and specific guidance in this area in the existing literature is sparse [6,10,13,14,15,16]. To understand the pre-HCT psychosocial services provided to patients and caregivers across Canada and inform pre-HCT psychosocial program development at our centre, we conducted an environmental scan of Canadian HCT programs. Environmental scans are used in health care settings to gain an understanding of current services and programs, assist with the quality improvement of programs, and inform program development [17].

## 2. Materials and Methods

### 2.1. Participants

Participants were psychosocial clinicians from HCT programs across Canada. Any psychosocial clinicians in such a program would have been eligible to participate. There were no exclusion criteria. 

### 2.2. Materials

See interview questions in Appendix A.

### 2.3. Procedure

Phase I: Recruitment

HCT program directors or hematologists within Canadian HCT programs were contacted by email to identify psychosocial contacts within their teams. A total of 13 Canadian HCT programs were identified and contacted.

Phase II: Contacting Psychosocial Clinicians

Identified psychosocial clinicians from Phase I at 13 sites were contacted by email with questions pertaining to their program (see Section 2.2). Psychosocial clinicians were invited to respond by email or arrange a telephone conversation for further discussion. Data were collected from April 2019 to May 2019. 

Phase III: Verification

This environmental scan was conducted as a program development initiative for our clinical service. Upon deciding to disseminate our findings, we determined that formal ethics approval was not needed because this quality improvement project was of minimal risk. However, we sought retrospective assent from our colleagues to use their information for this updated purpose. 

From July to October 2021, we emailed participants from the 10 sites that initially participated to provide an opportunity to complete missing items, confirm the accuracy of information, and offer an opportunity to withdraw. We also recontacted the 3 sites that had not responded with an additional invitation to participate. New participants were asked to report on their programs as of Spring 2019, the same time period as previous participants and prior to changes due to the COVID-19 pandemic.

### 2.4. Data Analysis

Author MQ conducted a qualitative content analysis following Vaismoradi and colleagues’ (2013) approach [18]. We approached the data with a realist and descriptive stance, and thus our coding was mostly semantic rather than latent. Data analysis steps included the following: (a) familiarizing ourselves with the data, (b) generating initial codes, taking into account frequency and relevance of responses, (c) searching for themes, (d) reviewing potential themes, (e) defining and naming themes, and, lastly, (f) producing the report. Several cycles of this process were undertaken with the team, in particular steps d to f. Establishing rigour in qualitative research depends on the method and theoretical framework [19]. In this study, we maintained credibility, dependability, and transferability in the following ways: peer debriefing (all authors), discussing fit between transcripts, codes, and themes (all authors), and keeping an audit trail of the research and analysis process (MQ). MQ also kept a reflexivity journal. We attempted to provide a description of our participants in Table 1 to allow readers to determine transferability (through this, the reader decides if the results are transferable to their community). However, due to the small number of HCT programs in Canada, all data were thoroughly de-identified.

## 3. Results

Psychosocial clinicians from 13 sites were contacted and 11 responded: 10 in the original recruitment and 1 additional clinician during verification. Participants were social workers (64%) and psychologists (36%). Participants responded via email (45%), phone (45%), and Zoom (10%). Of note, there is a small number of transplant programs across Canada, and patterns of reporting across questions may have rendered sites identifiable. Therefore, instead of using an identifier per site, we have reported the number of participants who have responded consistently, e.g., the notation 1P indicates reported by one participant, 2P denotes two participants, and so on. As such, the number of participants who endorsed each statement should not be taken as an indication of ‘strength of evidence’ but rather a continuous effort to show that data are coming directly from participant quotes rather than extensive researcher interpretation (in line with content analysis). We also elected to paraphrase our results rather than use direct quotes for the same reason. Results include four overarching themes, as shown in Table 2 below.

### 3.1. Psychosocial Team Composition

#### 3.1.1. Generalist versus Tumour-Specific Team Structure

Participants described the psychosocial staff available to pre-HCT patients at their sites as either a generalist (i.e., providing service to patients across tumour groups including HCT) or dedicated to the HCT service. Those with a generalist role typically had access to only social work (3P) except one site (1P) that listed a greater variety of psychosocial staff available to their whole centre. Four participants (4P) reported dedicated social workers for transplant patients, often at least one for inpatients and one for outpatients, with some reporting up to three or four. Two sites mentioned dedicated HCT psychology (2P) and one reported dedicated spiritual care (1P) in addition to dedicated social work. Five sites reported psychiatry was available by consult or referral, and two sites reported additional services available once patients were discharged through another service.

#### 3.1.2. Demands of Psychosocial Care on Social Workers

Only two participants reported a dedicated HCT psychologist, and none reported a dedicated psychiatrist (as shown in Table 1). One participant stated they did not have either discipline available to outpatients for many years (1P). Social workers, on the other hand, were the most commonly mentioned staff available to HCT patients and caregivers, and tended to be available in higher numbers than all other psychosocial staff. At the same time, at least four participants (4P) mentioned high demands on social workers, such as being responsible for the entire hematology unit or having high caseload demands elsewhere. The sentiment of only being consulted in high-risk situations or for crisis management, and being too extended to offer counselling or to better support HCT patients, was shared by several participants (3P). 

### 3.2. Criteria for Assessing Select HCT Patients

There were three sites that reported assessing all pre-HCT patients for psychosocial needs. All other sites assessed only subsets of transplant patients and each developed different criteria and processes for how to prioritize psychosocial resources.

#### 3.2.1. Psychosocial Needs and Risk

For many sites, the choice of which HCT patients to assess was made based on perceived psychosocial risk factors (4P) or Screening for Distress scores (1P) [20]. For example, two sites reported that an assessment was completed if referral was received from team members that identified higher psychosocial need or risk, regardless of the type of transplant (2P). Another explained that ‘high risk’ referrals to social work commonly included concerns with supports, mental health, behaviour, addictions, cognition, housing, or finances (1P).

#### 3.2.2. Transplant Type and Hospital Provision of Service 

For other sites, decisions about which HCT patients would receive pre-transplant psychosocial assessments were based on first the type of transplant, and second whether patients would be receiving HCT in-house or travelling to another hospital (see Table 3 below). Eight sites provided treatment for all transplant types, whereas three sites only treated (and thus assessed) their auto patients, sending allos to neighboring cities or provinces (2P). Another site specified that autos and allos from related donors stayed in-house while allos from unrelated donors travelled to another hospital. Presumably, psychosocial care would be assessed and addressed at the treating hospital, with the only noted exception being if the secondary hospital sent back a post-HCT referral for psychosocial care (1P). Several of these same sites clarified that they conduct a very brief assessment of practical and financial needs for most patients, providing further care only to the transplant patients they would be treating at their hospital (2P). In summary, patients being treated locally were also assessed based on local criteria. Those who were not being treated locally often received a brief assessment or may have been assessed elsewhere.

### 3.3. Components and Processes of Pre-HCT Assessments

#### 3.3.1. Common Components of Pre-HCT Assessment

Participants explained that their assessment questions were quite flexible in responding to patients’ priorities while also following a similar line of questioning for most patients (similar to semi-structured interviewing). Two participants elaborated on the areas they tried to ask about, which included the following: availability and support of family and loved ones especially post-discharge, coping, religious and spiritual beliefs, power of attorney, completion of will, encouragement to include loved ones in these discussions, financial worries, benefits, medication coverage, accommodations for out of town patients, understanding of medical plan, preparation for long hospital stays, experience with anxiety and depression, physical needs (e.g., homecare), general medical history, and community agencies involved in care (2P). Although some of the content areas overlapped in responses, most participants did not elaborate to this extent; thus, it is not clear what the most common assessment domains were. For example, some described a 90 min assessment with a social worker (1P) and others simply spoke of the use of distress screening tools by nurses (1P).

In addition, there appeared to be heterogeneity in terms of who was part of the assessment, for example, one participant described the assessment as an interdisciplinary meeting where the social worker joined the medical team (1P). Some described the assessment as a one-on-one meeting with the patient, and social worker (1P), or with a caregiver also present (1P), but most did not elaborate on the length of time or depth of discussion during assessments. In summary, although there were areas of overlap in assessment components, based on the data collected, there also appeared to be significant heterogeneity.

#### 3.3.2. Fluid Process for Biopsychosocial Understanding

All participants said they did not conduct a standardized assessment to evaluate pre-HCT psychosocial needs. Two mentioned using standardized or adapted standardized assessment tools as general guides for discussion (2P; i.e., not standardized administration). The tools used in this process were a set of questions developed by colleagues (1P) and the Stanford Integrated Psychosocial Assessment for Transplant (SIPAT), adapted for bone marrow transplant (1P). For the most part, participants guided their assessment using their clinical skills, the needs of the patient, and their knowledge of biopsychosocial areas of concern during the transplant process.

### 3.4. Design of Patient Education Sessions

In addition to each site’s varied approach to the pre-HCT assessment, some offered complementary or mandatory patient education sessions. These ranged from providing written material (1P) to extensive multi-day interdisciplinary sessions for patients and caregivers (1P). One site alternated their offerings by month between allo and auto patients (1P), and another offered individual education meetings with social work (1P). All four sites that reported offering education offered these in addition to individual assessments, where two of these assessed all patients and two assessed select patients.

## 4. Discussion

Our team conducted a national environmental scan to better understand current practices for assessing and preparing pre-HCT psychosocial needs of patients and caregivers. The 11 sites that participated reported variability in psychosocial team composition and disparate processes for pre-transplant psychosocial assessments and the provision of educational content. Our results highlight that there is significant heterogeneity and possibly little consistency in pre-HCT psychosocial processes across Canada.

These results show that there is a lack of common practices across programs, and, in fact, no two participants reported substantial similarities in any of the identified domains. Staffing varies widely from a minimum of two social workers and no other psychosocial clinicians available, even by consult, to interdisciplinary psychosocial teams dedicated to HCT. Assessment procedures vary from referrals for patients at perceived risk to the assessment of all pre-HCT patients. Although the limited literature recommends using standardized screening tools for psychosocial assessment prior to HCT (e.g., PAIC-HSCT, [21]; PACT, [22]; SIPAT, [6,23]), none of the sites surveyed reported using a HCT-specific screening tool. Only one site reported using Screening for Distress to guide referrals for psychosocial support. Incidentally, this is likely an underestimation of use since screenings for distress measures are a standard of practice in Canada [21]. Assessment content was most often guided by patient needs and clinical expertise without the inclusion of standardized administration of validated measures. Time and resource pressures likely contribute to the variety. We can also hypothesize that pressures such as organizational structure at different sites, historical and current budgetary decisions, and governmental and institutional priorities may have contributed. As well, there is negligible guidance in the existing academic literature, further inhibiting the ability to build evidence-based programs designed to best meet these needs. Without the literature to guide program development, we cannot know the pros and cons of these different approaches, or how to best assess and address psychosocial needs within time and resource limitations. 

**Figure 1:**
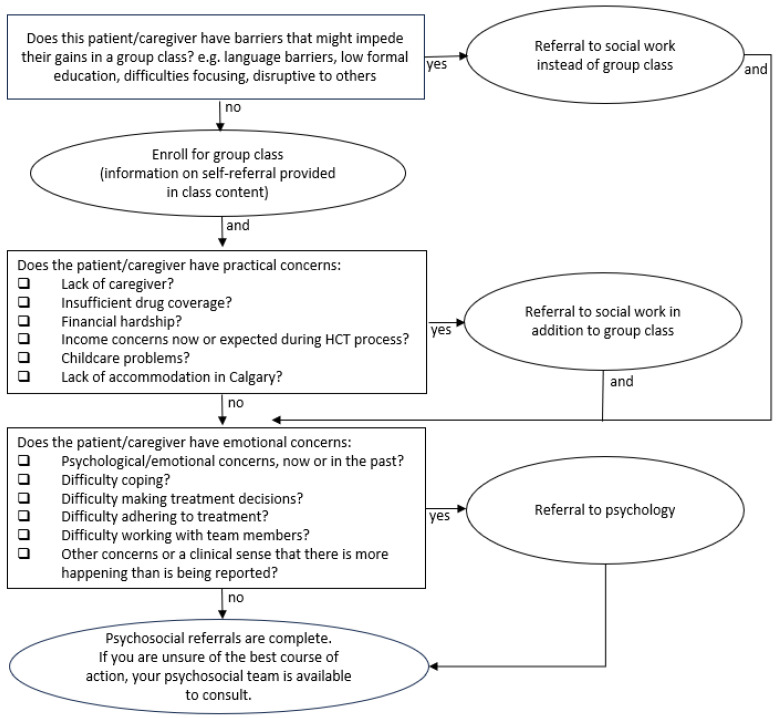
Stepped-Care Referral Pathway.

Further, there was considerable variability in practices used to identify candidates for pre-transplant HCT assessment and education. Some sites relied on referrals from medical team members, others assessed based on transplant type, and others assessed all patients. We cannot comment on the strengths of any of these approaches and wonder about the balance between maximizing resources for appropriate need versus patients with high psychosocial morbidity being unidentified and missed. With limited resources, it is important to achieve a balance between distributing limited resources for the appropriate needs of all patients awaiting HCT versus only prioritizing patients with higher identified psychosocial risk (e.g., patients with previous mental health concerns, patients expressing high levels of current distress, and patients with significant financial toxicity). Perhaps a stepped-care approach may address each side of this balance, such as offering a resource-efficient educational class to all patients and caregivers, taking self-referrals from patients and caregivers after the class for individual meetings, and relying on team referrals to identify other patients and caregivers who may need more intensive care. Please see Figure 1 for a suggested referral pathway for future consideration. Such referrals can be guided by Screening for Distress measurement (see [24] for an example of a screening for distress tool used by our centre at the time of data collection), which is a standard of practice in Canadian cancer care and elsewhere internationally. Our environmental scan demonstrates that each centre makes efforts to assess and address psychosocial needs pre-HCT. Identifying patients pre-HCT at risk for higher psychosocial distress allows for early intervention by psychosocial clinicians and may also improve clinical outcomes. For example, psychosocial risk and poor coping have been associated with higher readmission rates following HCT [25]. 

Among the different approaches reported, there could be various strengths that each approach offers. At this time, we can only report on them and cannot offer evidence for the advantages or disadvantages of each approach. Generalist programs (that serve all cancer care types and are not dedicated to HCT only) may be able to offer more variety of staff than programs with clinicians dedicated to teams. For example, dedicated HCT clinicians offer disease- and treatment-specific expertise, which arguably may better fit the needs and better inform the development of HCT programming. It is more expensive than a generalist program, which may be able to hire a social worker, a psychiatrist, and a psychologist, offering three disciplines to all patients and teams in their larger centres but may spread service more thinly. Or, conversely, perhaps a mix of some generalist and some dedicated clinicians may serve programs best. Another consideration arises from the theme of Demands of Care on Social Workers. Consistent with what can often be seen anecdotally in health care, our participants report that they have high clinical demands and often do not have access to support from other psychosocial disciplines. Some of our participants reported focusing on demands that are most pressing, such as crisis intervention, to the detriment of other needs that might impact outcomes, such as psychotherapy. As well, not all social workers are trained counsellors and not all social work job descriptions include psychotherapy within their scope. While hiring additional psychosocial clinicians may be fiscally aversive or impossible, there may be a toll on patients and existing team members. In the case of our social work participants, if covering for the omission of other psychosocial clinicians, they may be precluded from practicing to their full scope by focusing mostly on crises, with unreasonable or unachievable demands, to address all psychosocial aspects of care. 

### 4.1. Limitations

Given that the original scope of this environmental scan was to inform our centre’s program development (see [26]), we did not have the depth of data that we realize retrospectively could have been helpful. This includes a fuller picture of generalist versus dedicated HCT staff composition; contextual data about the size of each site, funding and resources, population size served; or how past experiences, program policies, or program evaluation led to the respective existing structures. Most participants did not indicate whether their education sessions were mandatory or optional. Finally, we did not have a clear definition of what “available by consult” or by referral might have meant at different sites (i.e., is a psychiatrist available by consult? a generalist team member? a consult-liaison psychiatrist for an overall centre or geographical region? or an external colleague who provides support as needed?) Had this environmental scan been developed with dissemination as an original goal, we would have had a better opportunity to define our questions and clarify responses.

### 4.2. Future Directions 

The most pertinent future direction we can derive from these findings is the evaluation of various approaches, considering effectiveness for patient-reported psychosocial outcomes, efficiency for patients, caregivers, and institutions, and the effectiveness of identifying and addressing need and risk. Some considerations include the following:

- What are patient experiences in attending classes, versus 1:1 assessments, versus obtaining support via self-referral, versus being identified and referred by team members?
- Does offering a stepped-care approach optimize the utilization of psychosocial services?
- What are the best ways to evaluate a stepped-care approach? Could they involve semi-structured interviews with patients, caregivers, and health care providers?
- Are there opportunities for interdisciplinary assessments with the medical team?
- Does lack of standardized administration of validated assessment tools contribute to inefficiency, difficulties allocating resources, and, potentially, psychosocial needs being missed?
- Could current non-standardized identification of patients with high psychosocial needs have inclusion or exclusion biases? For example, could minority populations be under-identified? Could stigmatized populations be over-identified? Are patients from diverse cultural backgrounds less likely to receive services based on differing cultural norms in asking for help, power differentials between patients and health care providers, or health literacy?
- The inclusion of patient and caregiver partners in future designs of research studies and educational programs is needed.

## 5. Conclusions

HCT programs across Canada have heterogeneous processes for assessing and addressing pre-HCT psychosocial needs. They vary in terms of team composition, the provision and content of psychosocial need assessment, and provision of HCT education materials. There are likely multiple factors that have contributed to disparate program development across Canada, and we are currently unaware of how these varied approaches may help or hinder psychosocial wellbeing, an important unaddressed need in HCT patients and their caregivers. Further research is needed to assess the efficacy and efficiency of these disparate approaches to help guide the evidence-based development of programs to support patients and caregivers in a manner that is sustainable across settings with their own resource pressures. This, in turn, will help to improve psychosocial and clinical outcomes.

## Figures and Tables

**Table 1:** Availability of three most frequently mentioned psychosocial staff in HCT.

Role		*n*	%
Social Worker			
	Dedicated to HCT teams	7	63.6%
	Available as part of generalist structure/available by consult	4	36.4%
	Not available	0	0%
Psychologist			
	Dedicated to HCT teams	2	18.2%
	Available as part of generalist structure/available by consult	2	18.2%
	Not available	7	63.6%
Psychiatrist			
	Dedicated to HCT teams	0	0%
	Available as part of generalist structure/available by consult	5	45.5%
	Not available	6	54.5%
Spiritual Care			
	Dedicated to HCT teams	1	9.1%
	Available as part of generalist structure/available by consult	3	27.3%
	Not available	7	63.6%

**Table 2:** Theme structure.

Psychosocial Team Composition	
	Generalist versus Tumour Specific Team Structure
	Demands of Psychosocial Care on Social Workers
Criteria for Assessing Select HCT Patients	
	Psychosocial Needs and Risk
	Transplant Type and Hospital Provision of Service
	Time and Resources
Components and Processes of Pre-HCT Assessment	
	Common Components of Pre-HCT Assessments
	Fluid Process for Biopsychosocial Understanding
Design and Components of Patient Education Sessions	

**Table 3:** Characteristics of pre-HCT psychosocial assessments.

Mode of Assessment			*n*	%
	All Patients are assessed		3	27.3%
	Select Patients are assessed			
		Allos only	1	9.1%
		All allos and autos with identified higher need	1	9.1%
		Autos only (allos are treated at a different centre)	1	9.1%
		All autos and allos with related donors (unrelated allos are treated at a different centre)	1	9.1%
		Those with high psychosocial needs/risk assessed	3	27.3%
		Most patient have a brief social work assessment for practical needs (not counselling)	1	9.1%
**Approach to Assessment**				
	Clinical conversation, not guided by standardized tool		9	81.8
	Clinical conversation, guided by standardized tool		2	18.1%
	Standardized tool used in standardized manner		0	0%
**Availability of Education Sessions**				
	Offered		4	36.4%
	Not offered		4	36.4%
	Data unavailable		3	27.3%

## Data Availability

For confidentiality reasons, further data will not be made available.

## Abbreviations

HCT	Hematopoietic Stem Cell Transplant (HCT)
Auto	Autologous
Allo	Allogeneic
SIPAT	Stanford Integrated Psychosocial Assessment for Transplant
PAIC-HSCT	Psychosocial Assessment Interview of Candidates for Hematopoietic Stem Cell Transplantation
PACT	Psychosocial Assessment of Candidates for Transplantation

## Appendix A

The following questions were posed to HCT practitioners in our survey:

(1) Who are the members of your BMT psychosocial team? (e.g., social work, psychology, psychiatry)
(2) Does your team offer pre-HCT psychosocial assessments/interviews?
  (a) Who are they offered to (i.e., all autos and allos, allos only, high risk individuals).
  (b) Is it a standardized assessment?
  (c) What questions are routinely asked?
  (d) Do you use any questionnaires or standardized assessment tools?
  (e) Is anything offered to individuals who do not get a pre-HCT psychosocial assessment?
(3) Do you offer group education classes, pre-transplant?
(4) Any Additional Notes you’d like to add?
